# Zinc- and Copper-Doped Mesoporous Borate Bioactive Glasses: Promising Additives for Potential Use in Skin Wound Healing Applications

**DOI:** 10.3390/ijms24021304

**Published:** 2023-01-09

**Authors:** Farzad Kermani, Simin Nazarnezhad, Zahra Mollaei, Sahar Mollazadeh, Alireza Ebrahimzadeh-Bideskan, Vahid Reza Askari, Reza Kazemi Oskuee, Ali Moradi, Seyede Atefe Hosseini, Zoleikha Azari, Francesco Baino, Saeid Kargozar

**Affiliations:** 1 Tissue Engineering Research Group (TERG), Department of Anatomy and Cell Biology, School of Medicine, Mashhad University of Medical Sciences, Mashhad 917794-8564, Iran; 2 Department of Materials Engineering, Faculty of Engineering, Ferdowsi University of Mashhad (FUM), Azadi Sq., Mashhad 917794-8564, Iran; 3 Department of Anatomy and Cell Biology, School of Medicine, Mashhad University of Medical Sciences, Mashhad 917794-8564, Iran; 4 Applied Biomedical Research Center, Mashhad University of Medical Sciences, Mashhad 917794-8564, Iran; 5 International UNESCO Center for Health-Related Basic Sciences and Human Nutrition, Mashhad University of Medical Sciences, Mashhad 917794-8564, Iran; 6 Department of Medical Biotechnology and Nanotechnology, Faculty of Medicine, Mashhad University of Medical Sciences, Mashhad 917794-8564, Iran; 7 Orthopedic Research Center, Mashhad University of Medical Sciences, Mashhad 917794-8564, Iran; 8 Institute of Materials Physics and Engineering, Applied Science and Technology Department, Politecnico di Torino, Corso Duca degli Abruzzi 24, 10129 Torino, Italy

**Keywords:** mesoporous bioactive glasses (MBGs), 13-93B3 borate bioactive glass, bioactivity, data science, tissue engineering, antibacterial properties, wound healing

## Abstract

In this study, zinc (Zn)- and copper (Cu)-doped 13-93B3 borate mesoporous bioactive glasses (MBGs) were successfully synthesized using nitrate precursors in the presence of Pluronic P123. We benefited from computational approaches for predicting and confirming the experimental findings. The changes in the dynamic surface tension (SFT) of simulated body fluid (SBF) were investigated using the Du Noüy ring method to shed light on the mineralization process of hydroxyapatite (HAp) on the glass surface. The obtained MBGs were in a glassy state before incubation in SBF. The formation of an apatite-like layer on the SBF-incubated borate glasses was investigated by X-ray diffraction (XRD) and scanning electron microscopy (SEM). The incorporation of Zn and Cu into the basic composition of 13-93B3 glass led to changes in the glass transition temperature (T_g_) (773 to 556 °C), particle size (373 to 64 nm), zeta potential (−12 to −26 mV), and specific surface area (S_BET_) (54 to 123 m^2^/g). Based on the K-means algorithm and chi-square automatic interaction detection (CHAID) tree, we found that the SFT of SBF is an important factor for the prediction and confirmation of the HAp mineralization process on the glasses. Furthermore, we proposed a simple calculation, based on SFT variation, to quantify the bioactivity of MBGs. The doped and dopant-free borate MBGs could enhance the proliferation of mouse fibroblast L929 cells at a concentration of 0.5 mg/mL. These glasses also induced very low hemolysis (<5%), confirming good compatibility with red blood cells. The results of the antibacterial test revealed that all the samples could significantly decrease the viability of *Pseudomonas aeruginosa*. In summary, we showed that Cu-/Zn-doped borate MBGs can be fabricated using a cost-effective method and also show promise for wound healing/skin tissue engineering applications, as especially supported by the cell test with fibroblasts, good compatibility with blood, and antibacterial properties.

## 1. Introduction

Over the last few years, numerous scientific attempts have been made to develop advanced therapies for managing hard-to-heal skin wounds, like diabetic ulcers [1]. Among the promising candidates, the bioceramics family (e.g., bioactive glasses (BGs)) have been mentioned as potential advanced therapies for both acute and chronic skin wounds [2,3,4]. Experimental research has indicated that BGs, as a highly versatile class of inorganic biocompatible materials, could accelerate tissue repair and regeneration [5]. The first invented BG (45S5 Bioglass^®^) was a silicate-based glass developed for managing hard tissue diseases and disorders [6,7,8]. Over the years, other formulations of BGs were developed that have exceptional biological activities for soft tissue healing, as well [9]. For example, borate-based glasses with excellent bioactivity and a high ion release rate are known to be the most recent members of the BG family with approved therapeutic properties for wound healing [10,11,12]. From a biological point of view, borate-based BGs are commonly doped with specific types of elements (e.g., copper and zinc) to improve their in vitro and in vivo behaviors, such as cytocompatibility, antibacterial performance, pro-angiogenic potency, regenerative capacity, etc. [13,14,15].

Traditionally, borate BGs are synthesized using the melt-quenching method, due to the inherent easiness of the process, but, on the other hand, the process needs specific equipment, such as high-temperature furnaces. Accordingly, the sol–gel method was gradually applied for the production of borate glasses with specific features, such as a porous texture, that makes them suitable candidates for drug delivery strategies [16,17,18]. However, expensive reagents (i.e., methoxy precursors) are generally required for producing sol–gel-derived borate glasses. On this matter, Kermani et al. successfully developed a modified sol–gel synthesis method for obtaining borate-based MBGs with a high cost–benefit ratio, in which a polymeric substrate (polyvinyl alcohol (PVA)) was used to stabilize boric acid in the reaction and to prepare nano-sized glass particles with a porous texture [16].

As a rule of thumb, specific kinds of therapeutic elements at defined concentrations are added to the basic composition of BGs in order to improve their inherent properties. In this regard, zinc (Zn) has been well-demonstrated to render anti-inflammatory and antibacterial activities to biomaterials without any significant adverse effects [15]. Another beneficial element is copper (Cu), which can improve the biological properties of BGs in favor of tissue engineering applications like enhanced antibacterial and pro-angiogenic properties [7]. It should be noted that high consumption of Zn and Cu elements can cause adverse effects in the body [19,20]. An excessive intake of Zn and Cu may cause toxicity (e.g., nausea and vomiting) and brain oxidative damage, respectively.

Synthesis of BGs, as well as the prediction of glass properties, can take advantage of computational approaches to develop more efficient materials [21,22]. Data science, an interdisciplinary field of science, is generally utilized to understand and analyze data and their correlations for the extraction or extrapolation of knowledge from different data, including noisy, structured, and unstructured information [23,24,25]. Data science algorithms have become an interesting issue for materials researchers, including glass scientists, seeking to understand composition–property relationships [26]. For example, Ouldhnini et al. recently utilized molecular dynamics simulations to investigate the elastic and structural properties of densified 45S5 BG and liquids over a wide range of densities [27].

In the current study, we examined the composition–property relationships in mesoporous Zn- and Cu-doped 13-93B3 borate BGs, which were synthesized using the sol–gel route in the presence of Pluronic P123. This study aimed to highlight the role of data science algorithms in the deep investigation of the physicochemical and biological properties of BGs. Based on data science algorithms, a new possible mechanism behind the excellent bioactivity of borate BGs was found and introduced. Furthermore, the biological properties of the prepared glasses were studied through cell viability, blood compatibility, and antibacterial tests in order to assess their potential in tissue engineering applications, with special focus on wound healing.

## 2. Results

### 2.1. Thermodynamic Characteristics of the MBGs

**Figure 1:**
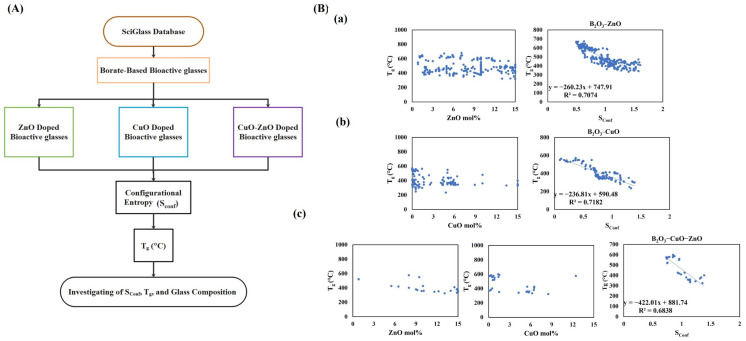
The decision tree for possible consequences of glass composition on T_g_ and S_Conf_ (**A**), and the relation of T_g_ with composition and S_Conf_ for B_2_O_3_–ZnO (**a**), B_2_O_3_–CuO (**b**), and B_2_O_3_–ZnO–CuO groups (**c**) (**B**).

The obtained data from the SciGlass database using the presented decision tree (Figure 1A) are shown in Figure 1B. The relation of the composition of S_Conf_-T_g_ of the bioactive borate-based glasses is shown in Figure 1B(a–c). According to Figure 1B(a), S_Conf_ and T_g_ had a linear relation with a regression coefficient of 0.71 in the B_2_O_3_–ZnO compositions. A similar regression coefficient was also found for the same couple of parameters in the B_2_O_3_–CuO group (Figure 1B(b)). In the B_2_O_3_–ZnO–CuO group, the regression coefficient was slightly lower (0.68), still indicating a linear correlation (Figure 1B(c)). According to the extracted data, the indirect relation between S_Conf_ and T_g_ was obvious among all the groups.

### 2.2. Physical Characterizations of MBGs

#### 2.2.1. Thermal, DLS, BET, and TEM Analyses

The obtained values for T_g_, particle size, zeta potential, and S_BET_ of the synthesized MBGs are presented in Table 1. Based on the thermal analysis, T_g_ values of the un-doped MBGs (773 °C) reduced after adding the dopants to the glass composition (679, 566, 556 °C recorded for Zn7.5, Cu7.5, and Zn/Cu7.5 groups, respectively). In addition, the incorporation of Zn, Cu, and Zn/Cu into the glass network significantly decreased the particle size (D_V_90) of the MBGs from 378 nm (B0) to 92 (Zn7.5), 96 (Cu7.5), and 64 nm (Zn/Cu7.5). The zeta potential values were significantly increased in the doped glasses, compared to the un-doped counterparts. The zeta potential values of the glass particles showed a significant increase (*p* < 0.05) after doping with Zn and Cu (from −12 to −18 and −26 mV, respectively); the Zn/Cu-co-doped MBGs had higher values of the zeta potential (in the range of −22 to −26 mV).

The S_BET_ data indicated an increasing trend from 54 m^2^/g for sample B0 to 131, 122, and 123 m^2^/g for the Zn7.5, Cu7.5, and Cu/Zn7.5 groups, respectively. According to the data, the S_BET_ of Zn-doped MBGs was significantly (*p* < 0.05) higher than its Cu-doped counterparts. Furthermore, the S_BET_ values of the Cu/Zn-co-doped MBGs were significantly higher than the MBGs doped with single dopants (see Table 1).

**Figure 2:**
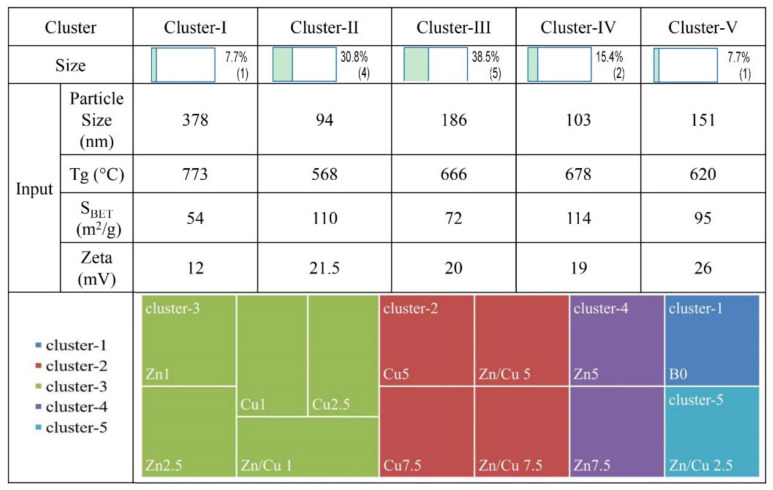
The classification of the MBGs based on thermal, DLS, and BET analyses.

The data collected in Table 1 were classified, and the results are also shown in Figure 2. The classification results divided the synthesized MBGs into five clusters, including cluster I-7.7% (B0), cluster II-30.8% (Cu5, Cu7.5, Zn/Cu 5, Zn/Cu7.5), cluster III-38.5% (Zn 1, Zn2.5, Cu1, Cu2.5, and ZnCu1), cluster IV-15.4% (Zn 5 and Zn7.5), and cluster V-7.7% (Zn/Cu 2.5). In the classification, each feature was given a 7.7% weight. It is important to note that K-means clustering is an unsupervised machine learning (ML) technique that involves splitting n input space into K clusters with each input belonging to the cluster with the closest mean (cluster center). In K-means clustering, the optimal K is determined by the elbow, silhouette, and gap statistics methods.

**Figure 3:**
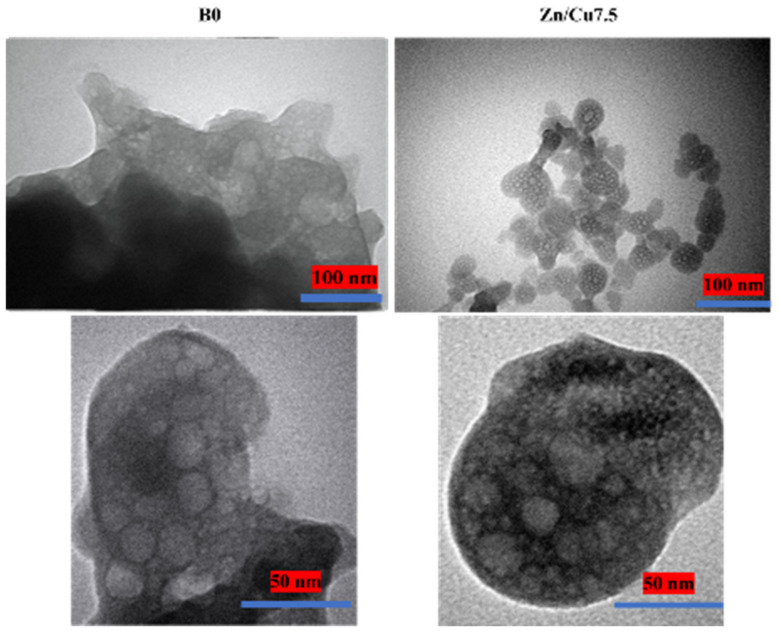
TEM images of dopant-free (B0) and doped MBGs with 7.5 mol% Zn/Cu (Zn/Cu7.5).

Figure 3 shows TEM micrographs of dopant-free and co-doped MBGs with 7.5 mol% Zn/Cu. The mesoporous nature of the MBGs can be seen in the TEM images. Furthermore, it was noted that the dopants reduced the particle size of the glass samples, in agreement with DLS results.

#### 2.2.2. SEM Analysis

**Figure 4:**
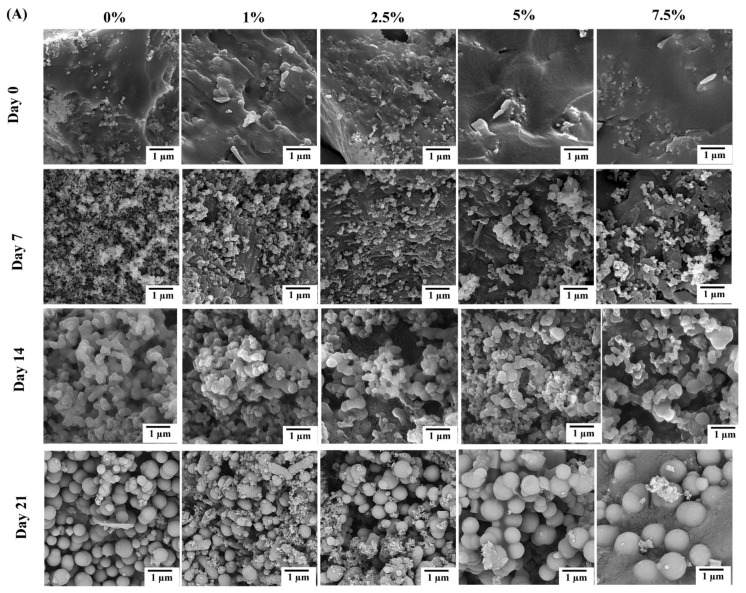

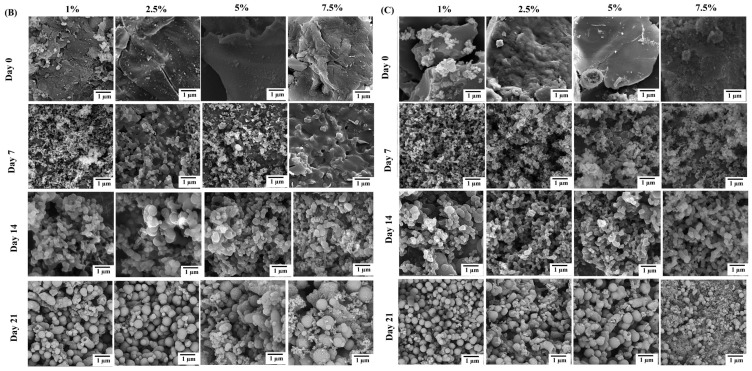
The SEM micrographs of the MBGs doped with Zn (**A**), Cu (**B**), and Zn/Cu (**C**) after 0, 7, 14, and 21 days of soaking in SBF.

The SEM micrographs of the MBGs doped with Zn, Cu, and Zn/Cu (also compared with dopant-free 13-93B3 MBG) are shown in Figure 4A–C, respectively. According to the images, the as-synthesized MBGs (Day 0) did not have any specific morphological features on their surface, with the particles exhibiting an irregular shape. HAp mineralization can be seen in the SBF-incubated MBGs after 7 days. Based on the micrographs, the new phase nucleated onto the glass surface grew over time, and a typical globular morphology was observed after 21 days of incubation of the samples in SBF.

#### 2.2.3. XRD Analysis

**Figure 5:**
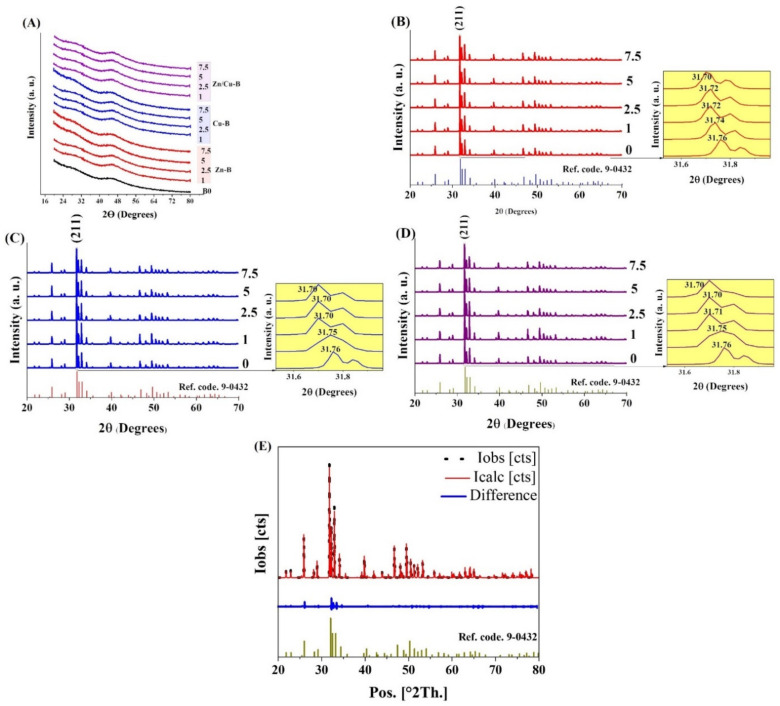
The XRD patterns of the synthesized MBGs before incubation in SBF (**A**) and the SBF-incubated MBGs with Zn (**B**), Cu (**C**), and Zn/Cu (**D**) after 21 days. The Rietveld refinement pattern of the B0 group after 21-days of immersion in SBF (**E**).

The XRD patterns of the synthesized MBGs (Figure 5A) confirmed the amorphous nature of all the samples (only the typical broad halo was visible). Figure 5B–D show the XRD graphs of the MBGs doped with Zn, Cu, and Zn/Cu after 21 days of immersion in SBF, respectively. Based on the data, crystalline hydroxyapatite (HAp) (Ref. code. 9-0432) was observed in all the samples. The calculated crystallinity degree of the mineralized HAp onto the un-doped MBG (B0) was about 80%. The crystallinity degree values of the mineralized HAp for Zn-, Cu-, and Zn/Cu-doped MBGs were in the range of 65–76, 63–74, and 60–71%, respectively. According to the results, the major diffraction peak corresponding to the (211) plane of HAp shifted to lower degrees in all the samples. According to Figure 5B–D, the 2θ position of (211) peak of HAp changed from 31.76° in the un-doped glasses to 31.70° in the samples containing dopants (7.5 mol%). The results of the calculation of the HAp lattice constants (a = b, c), which were extracted from the Rietveld refinement results, are presented in Table 2. Furthermore, the Rietveld refinement analysis results of the B0 group, including observed (obs), calculated (calc), and difference (obs-calc) intensities, are illustrated in Figure 5E. According to the data, the lattice constant of HAp (a = 9.424 Å, c = 6.879 Å) increased to (a = 9.441 Å, c = 6.89 Å), (a = 9.441 Å, c = 6.88 Å), and (a = 9.446 Å, c = 6.891 Å) in the doped MBGs with 7.5 mol% of Zn, Cu, and Zn/Cu, respectively.

### 2.3. Bioactivity Assessment

#### 2.3.1. Tensiometry

**Figure 6:**
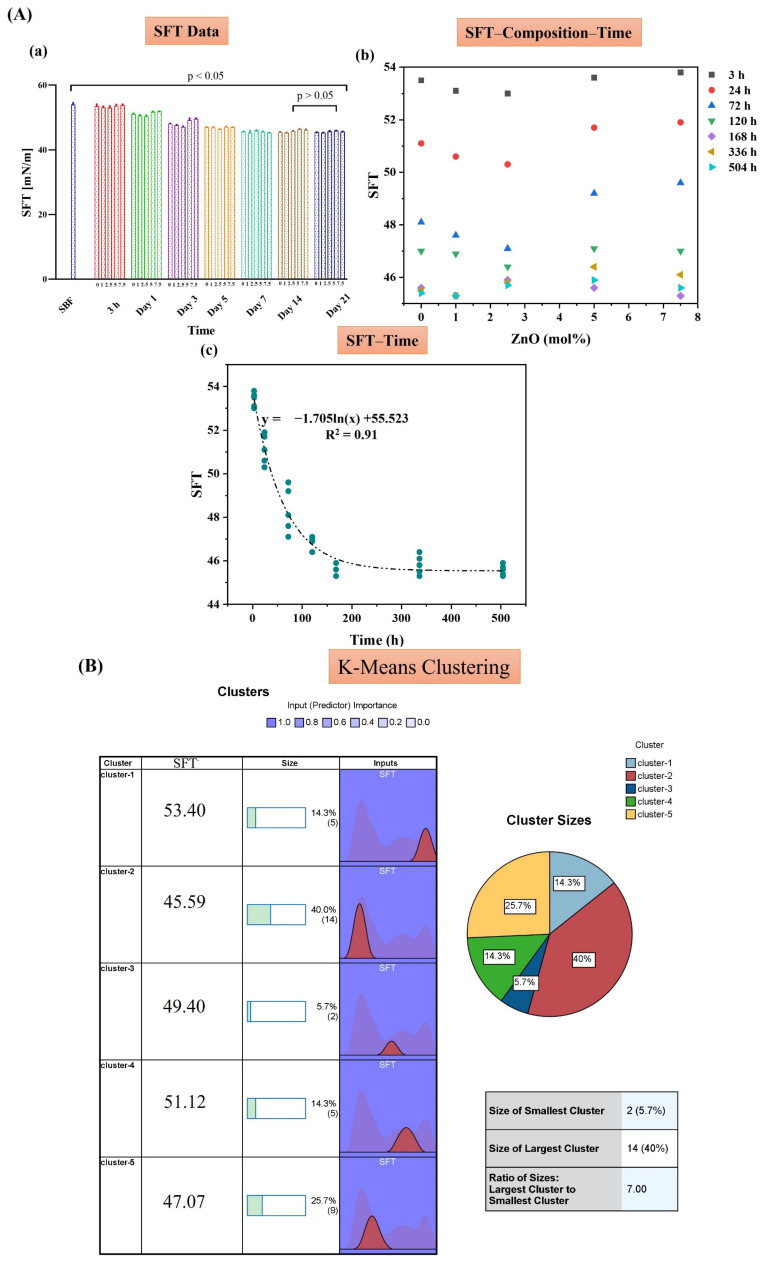

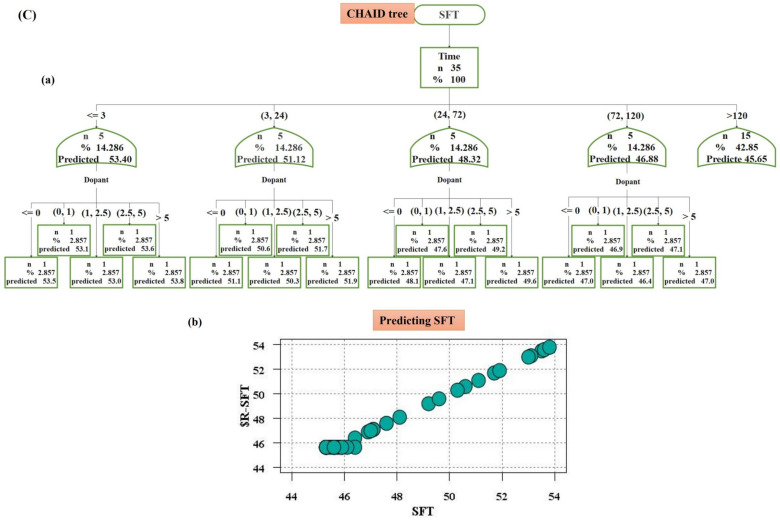
The SFT data of the Zn-doped MBGs group, including SFT data (**a**), the SFT–time–composition graphs (**b**), and the fitted ln-type graphs of SFT–time (**c**) (**A**). The K-means clustering classification of the data (**B**). The CHAID tree (**a**) and the relation of the predicted and experimental SFT (**b**) (**C**).

**Figure 7:**
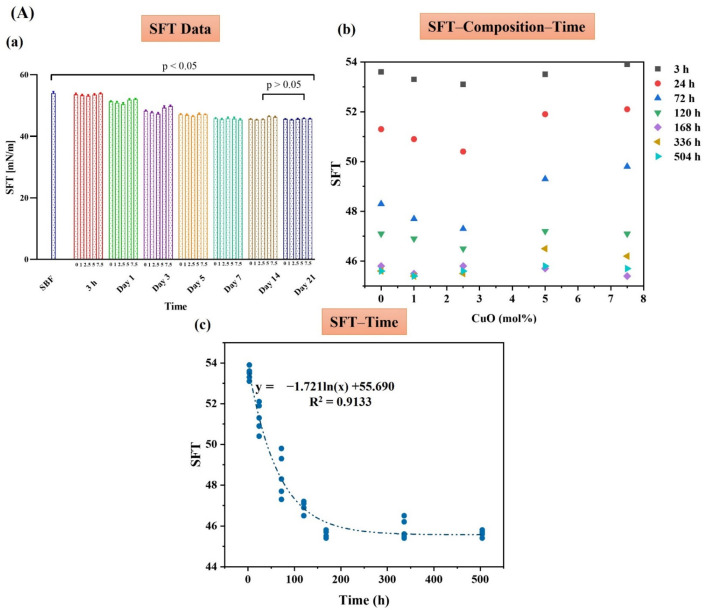

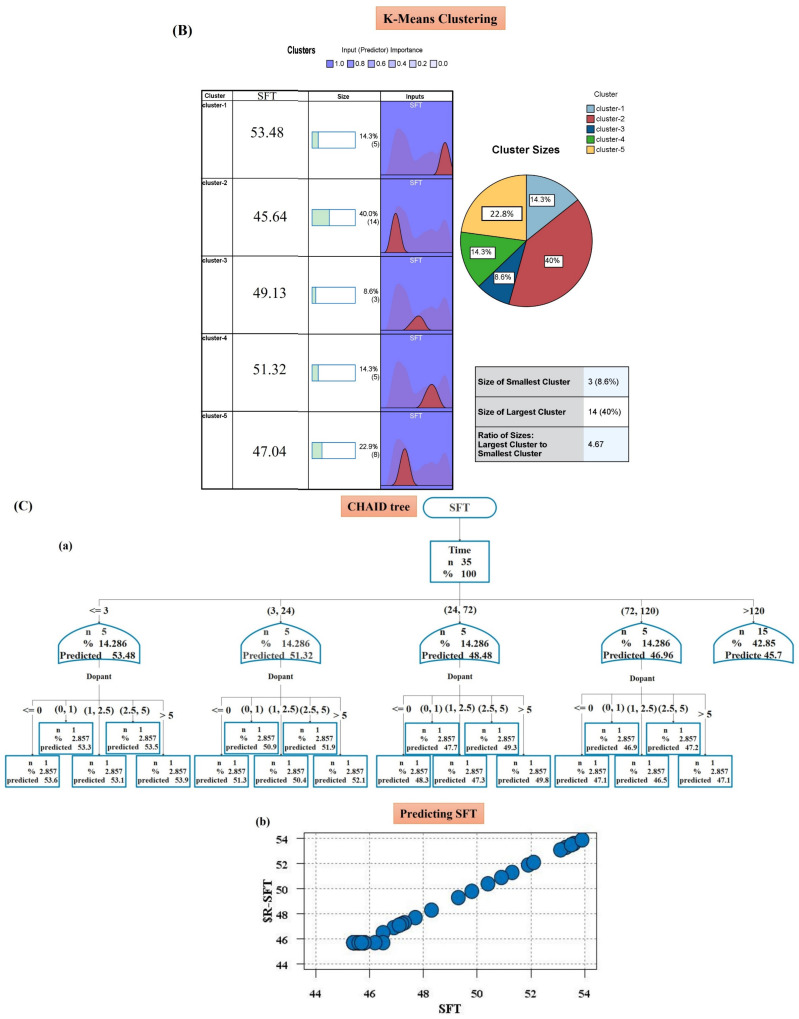
The SFT data of the Cu-doped MBGs group, including SFT data (**a**), the SFT-time-composition graphs (**b**), and the fitted ln-type graphs of SFT-time (**c**) (**A**). The K-means clustering classification of the data (**B**). The CHAID tree (**a**) and the relation of the predicted and experimental SFT (**b**) (**C**).

**Figure 8:**
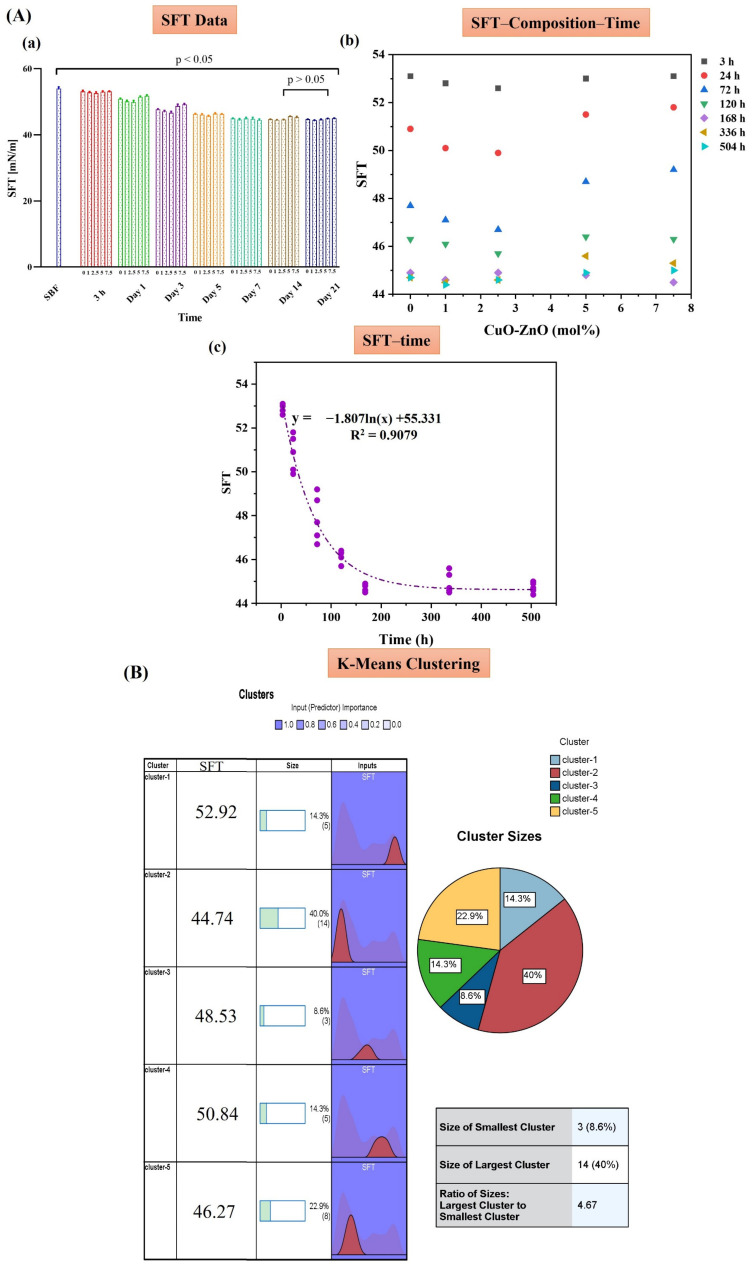

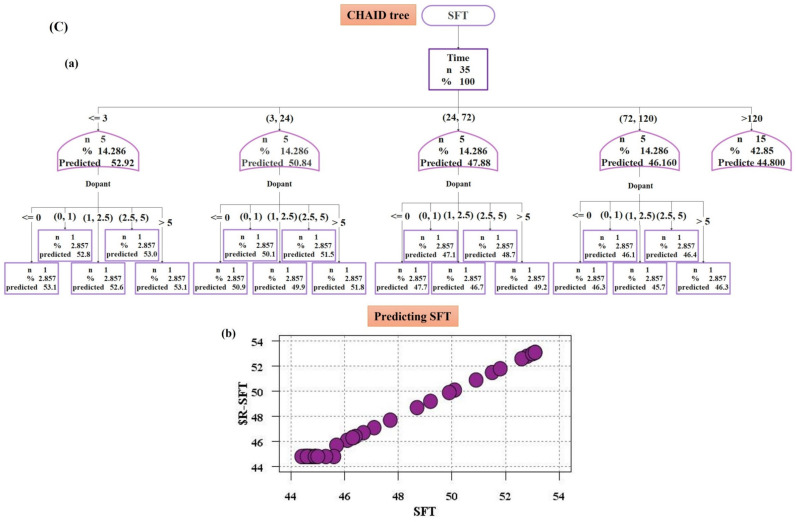
The SFT data of the Zn/Cu-doped MBGs group, including SFT data (**a**), the SFT–time–composition graphs (**b**), and the fitted ln-type graphs of SFT–time (**c**) (**A**). The K-means clustering classification of the data (**B**). The CHAID tree (**a**) and the relation of the predicted and experimental SFT (**b**) (**C**).

The results of SFT of the MBGs doped with Zn, Cu, and Zn/Cu are presented in Figure 6, Figure 7 and Figure 8, respectively. The data of each figure are divided into three sections, including (A) the raw data of SFT at different times, the graph of SFT–Composition–Time, and the relation found between SFT and time in SFT–time graphs. In section (B), the results of the K-means clustering algorithm of the data are presented. In section (C), the CHAID tree of the data, and the relation between the predicted SFT with the CHAID tree and the experimental data, are presented. Based on the data, in all the groups, i.e., Zn-MBGs (group I), Cu-MBGs (group II), and Zn/Cu-MBGs (group III), the logarithmic (ln-type) graphs fitted SFT–time curves with a regression coefficient higher than 0.9. According to the K-means clustering algorithm, all the groups were classified into five clusters. According to the CHAID tree, the SFT–time–composition data were divided into five numeric ranges, including ≤3 h, (3–24) h, (24–72) h, (72–120) h, and ≥120 h in the MBGs developed in the current research. Specifically, the five clusters of data, based on composition, are the un-doped MBGs, the doped samples in the range of (1–2.5) mol%, (2.5–5) mol%, and ≫5 mol%. Based on the data, it was possible to predict SFT values, based on the composition and time, with an accuracy higher than 90% in all the groups.

Based on the data presented in Figure 6, Figure 7 and Figure 8, section A(a), we proposed an index (β) for “quantifying” bioactivity of the MBGs in Table 3. The values of β were calculated based on the variation of SFT over time (SFT_t_) compared to the first calculated amount of SFT (SFT_0_) (control) (Equation (1)). Since SFT_t_ might be higher than SFT_0_, in such cases, it is appropriate to use the absolute value function:

(1) $$\beta \;=\sum^{\;}\left|\frac{{\mathrm{SFT}}_{0}-{\;\mathrm{SFT}}_{T}}{{\mathrm{SFT}}_{0}}\right|$$

Table 3 shows that the normalized β number of MBGs doped with 1, 2.5, 5, and 7.5 mol% of Zn changed by 5, 4.8, −7.5, and −7.8, compared to the control, correspondingly. In the Cu-doped group, the values were 4.6, 2.8, −8.8, and −9.6, respectively. The values for the co-doped group were 17.8, 15.2, 3.1, and 2.4, respectively.

#### 2.3.2. The pH Measurement and Ion Release Profile

**Figure 9:**
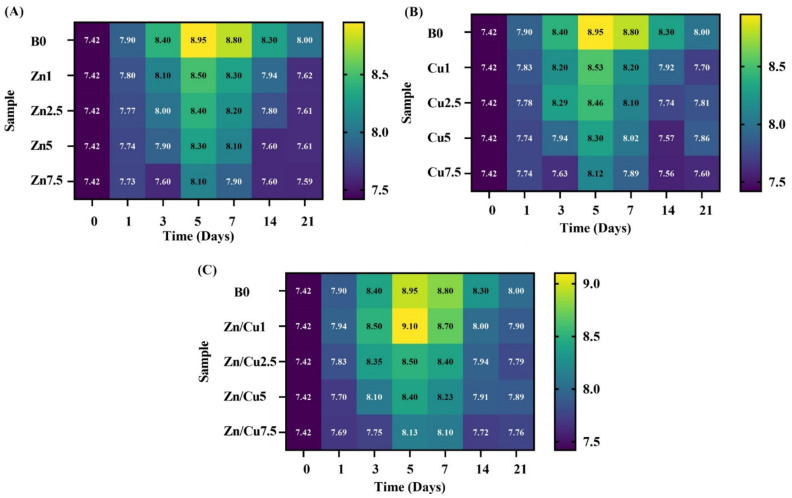
The color mapping profile of the pH-time variation for the doped MBGs with Zn (**A**), Cu (**B**), and Zn/Cu (**C**).

The pH color mapping profiles of the pH-time variation of the MBGs doped with Zn, Cu, and Zn/Cu are illustrated in Figure 9A–C, respectively. According to the maps, the pH of all MBGs showed an increase for up to the first five days, then the values decreased.

**Figure 10:**
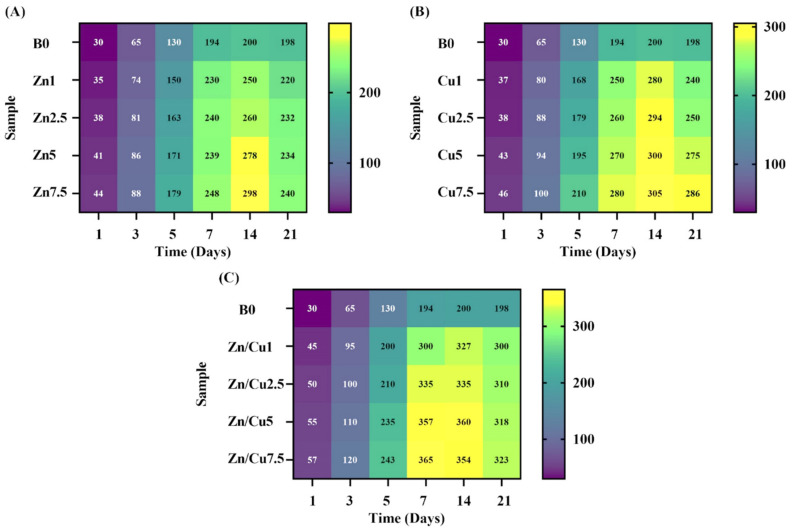
The color mapping of the boron (**B**) release profile for the MBGs doped with Zn (**A**), Cu (**B**), and Zn/Cu (**C**).

Figure 10A–C show the color mapping of the boron (B) release profile from the doped MBGs with Zn, Cu, and Zn/Cu, respectively. According to the maps, the release of B continually increased in all the groups for up to the first two weeks. After day 14, a decreasing trend was seen for the release of B^3+^ ions. The highest measured concentration for B^3+^ ions in the un-doped MBGs was 200 ppm. Moreover, the highest values for the B^3+^ ions concentration in the doped MBGs with Zn, Cu, and Zn/Cu were 298, 305, and 365 ppm, respectively.

**Figure 11:**
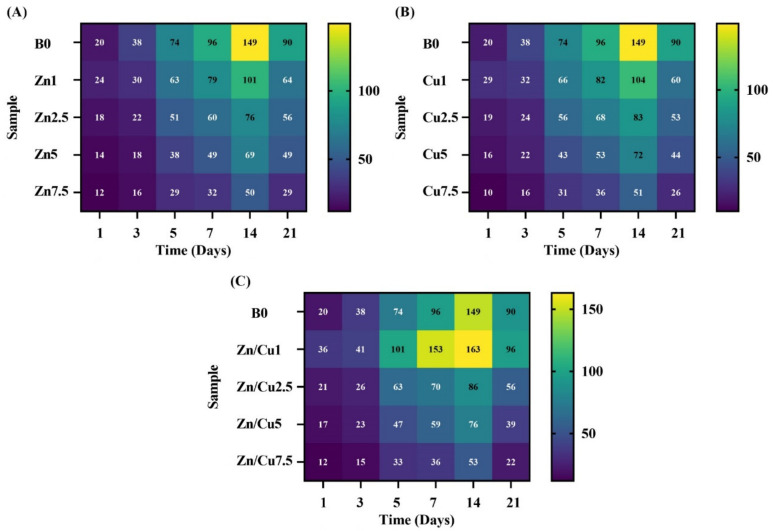
The color mapping profile of Ca-release for the doped MBGs with Zn (**A**), Cu (**B**), and Zn/Cu (**C**).

The color maps of the calcium (Ca) release profile for the glasses doped with Zn, Cu, and Zn/Cu are shown in Figure 11A–C, respectively. Regarding the maps, the release concentration of Ca exhibited an increase for all the SBF-soaked samples for up to the first 14 days. The release of Ca^2+^ ions from the doped samples showed a decrease, as compared to the un-doped counterparts, except for the Cu/Zn–co-doped MBGs with 1 mol% Zn/Cu. The highest measured concentration of Ca^2+^ ions was 163 ppm for the mentioned samples after 14 days of immersion.

### 2.4. Biological Evaluations

#### 2.4.1. Cell Viability

**Figure 12:**
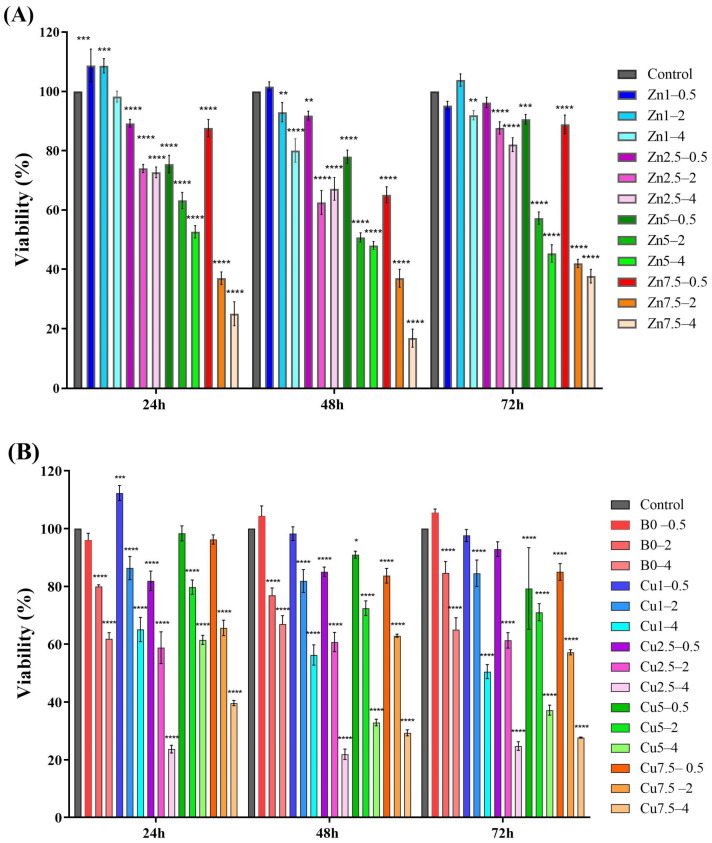

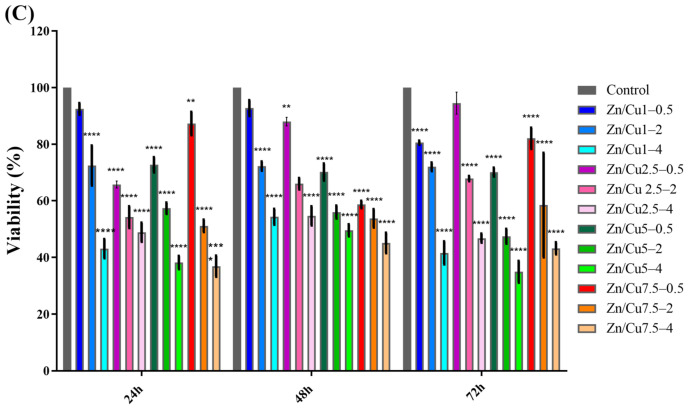
The effect of the borate MBGs doped with Zn (**A**), Cu (**B**), and Zn/Cu (**C**) on the viability of L929 cells after 24, 48, and 72 h. The significant changes were marked, based on *p* values (* *p* < 0.05, ** *p* < 0.01, *** *p* < 0.001, and **** *p* < 0.0001).

The effects of the Zn- and Cu-doped MBGs on the viability of L929 cells are shown in Figure 12, revealing that the cell viability improved in the groups treated with the glasses doped with 1 mol% of Zn (Zn1) (0.5 and 2 mg/mL) and 1 mol% of Cu (Cu1). However, the cell viability decreased along with increasing the dopant concentrations in all the groups at different incubation intervals (24, 48, and 72 h).

#### 2.4.2. Blood Compatibility

**Figure 13:**
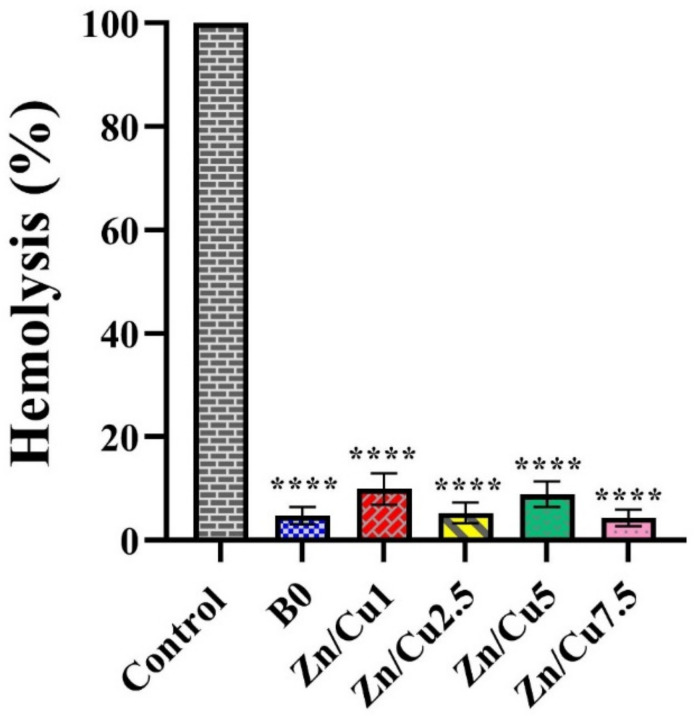
The effect of MBGs on hemolysis of red blood cells. The significant changes were marked based on *p* values (**** *p* < 0.0001).

Figure 13 displays the outcome of the blood compatibility assay for the prepared MBGs. The hemolysis percentage of all the samples was significantly (*p* < 0.0001) lower than the positive control group (hemolyzed blood with distilled water). Based on the results, the amounts of hemolysis for the un-doped MBGs and those doped with 1, 2.5, 5, and 7.5 mol% of Zn/Cu were 4.77 ± 1.36, 9.94 ± 2.49, 5.31 ± 1.63, 8.9 ± 1.98 and 4.3 ± 1.35%, respectively.

#### 2.4.3. Antibacterial Activity

**Figure 14:**
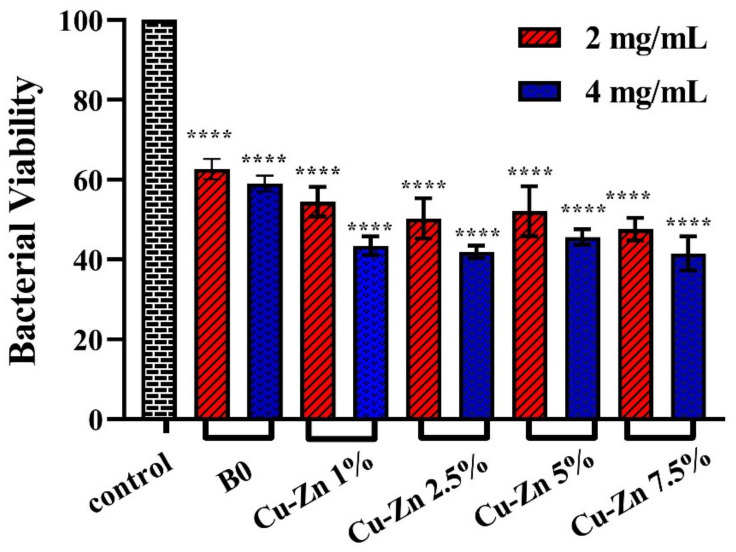
The antibacterial activity of the borate MBGs co-doped with Zn and Cu against *P. aeruginosa* strains after 24 h. The significant changes were marked based on *p* values (**** *p* < 0.0001).

The results of the antibacterial activity of the MBGs with concentrations of 2 and 4 mg/mL are shown in Figure 14, showing that the bacterial viability was significantly reduced after incubation with different glass samples for 24 h. However, the MBGs doped with 1 mol% of Zn/Cu (Zn/Cu1) had the greatest reductive effect on the viability of *P. aeruginosa* bacteria (54.47 ± 3.01% and 43.45 ± 1.91% for concentrations of 2 and 4 mg/mL, respectively). Furthermore, based on the statistical data, the viability of the treated bacteria with the doped MBGs were significantly lower than those of the dopant-free MBG counterparts.

## 3. Discussion

In the present study, 13-93B3 borate-based MBGs were doped with 1–7.5 mol% of Zn and Cu (Table 4) to develop suitable substances for potential use in tissue engineering strategies. In the sol–gel synthesis process, the mesoporous glass particles were obtained in the presence of Pluronic P123 as a structure-directing agent. It is well-known that adding dopants to the BG composition can affect the glass network (e.g., bond length and concentration of non-bridging oxygens (NBOs)) as well as glass physicochemical and biological properties [22,28]. To investigate thermodynamic aspects of the synthesized MBGs, initially, the available data of bioactive borate glass containing Zn and Cu was extracted from the SciGlass database. To draw a correct relation between chemical composition and the glass properties, the S_Conf_ of the extracted data was calculated using Equation (3) (see Section 4.2). It is reported that S_Conf_, as a thermodynamic variable, is correlated to glass properties, including T_g_, melting point, and concentration of NBOs [22,28]. Adding dopants to the glass network increases the bond length, ion mobility, and concentration of NBOs [29,30]; thereby, the glass network “disruption” increases, yielding the increment of S_Conf_. As shown in Figure 1, a negative linear relation between S_Conf_ and T_g_ could be observed for the BGs doped with Zn, Cu, and Zn/Cu.

According to the data reported in Table 1, Zn had a greater effect on T_g_ (evidenced in a change from 773 to 566 °C) as compared to Cu (evidenced in a change from 773 to 676 °C). Possibly, this effect was due to the slightly larger ionic radius of Zn^2+^ (0.74 Å) than Cu^2+^ (0.73 Å). In this way, the Zn–O bond strength was lower than that of Cu–O, which affected the chaining of the glass network and T_g_. In the co-doped systems, T_g_ decreased from 773 to 556 °C. Consistent with the literature [17], the greatest changes in T_g_ were expected for the Zn/Cu co-doped MBGs, as they have higher network disordering due to the high ionic repulsion force of the ions.

Based on the K-means algorithm, all the characterizations data (Table 1) are classified into five clusters (Figure 2). This data clustering was in line with previous reports [31,32], which indicated the role of dopants on the particle size, surface charge, and surface area of the MBGs. Based on the obtained results, adding and increasing the concentration of the dopants led to the following: (I) a decrease in the particle size (from 378 to 64 nm), due to the role of the dopants in the prevention of particle growth and agglomeration; (II) decrease in the zeta potential value (from −12 to −26 mV), as a result of the gradient distribution of dopants from bulk to the surface, as also observed in [16,33]; (III) and enhancement of the S_BET_ (from 54 to 131 m^2^/g), because of the important role of dopants on particle size of MBGs (the lower the particle size, the higher the S_BET_). For clarification of the mentioned results, we used TEM analysis. The TEM images in Figure 3 demonstrated the mesoporous nature of the particles, as well as the decrease in particle size. These results, and the K-means clustering data, indicated that the most important concentrations of the doped elements were 1 and 5 mol%, which had independent data in the sol–gel synthesized borate glasses.

SEM (Figure 4) and XRD (Figure 5) analyses clarified the mineralization of borate glasses in SBF, showing the growth of the HAp-like layer with spherical morphology onto all the synthesized MBGs. HAp with a high degree of crystallinity (65–80%) was mineralized after 21 days of SBF incubation. These values were significantly higher than the crystallinity degree of mineralized HAp in the silicate glasses [17,18,34]. A minor shift of the diffraction peak, corresponding to the (211) plane of Hap, and an increase of lattice constants of Hap, were observed with increasing of the dopant concentration (Table 2). This observation might be associated with the doping process of Zn^2+^, Cu^2+^, and B^3+^ ions into the HAp structure during the adsorption/desorption process of ions in SBF, as reported elsewhere [16]. Changes in the dynamic surface tension (SFT) of SBF-immersed glasses, including Zn-doped (Figure 6), Cu-doped (Figure 7), and Zn/Cu-doped MBGs (Figure 8), were evaluated for up to 21 days. According to the data, we suggested a new explanation for the mineralization of HAp on the surface of these borate MBGs, in addition to previously proposed scientific reasons (e.g., change in the pH of SBF and ion saturation) [35,36]. We found that during the adsorption/desorption process of ions in the SBF, the SFT of SBF changed continuously. In agreement with the known mechanisms for nucleation and growth at liquid–solid interfaces [37,38], these changes might promote HAp nucleation at the liquid–solid interface. On the basis of SFT data, we calculated β as a new criterion of bioactivity (Table 3). Bioactivity measurements based on this criterion indicated that co-doped MBGs with 1 mol% Zn/Cu (Zn/Cu1) had 17.8% higher bioactivity than their dopant-free counterparts. The current data indicated that the bioactivity of the doped groups decreased with increasing amounts of dopants; the normalized β compared to B0 MBGs decreased from 5 to −7.8 in Zn-doped MBGs, from 4.6 to −9.6 in Cu-doped MBGs, and from 17.8 to 2.4 in the co-doped group. These phenomena can be explained by changes in the solubility of the glass caused by chemical bonds between Zn–O/Cu–O and Ca–O, as discussed in [17,34]. According to the K-means clustering algorithm and CHAID tree results, the appropriate time for the evaluation of bioactivity of the sol–gel synthesized borate-based MBGs could be considered in the range of 3–168 h, including 3 h, 24 h (1 day), 72 h (3 days), 120 h (5 days), and 168 h (7 days). Accordingly, it may be interesting for the community to investigate the changes in the SFT of SBF containing other types of BGs (e.g., silicate-based BGs) to provide an in-depth view of the significance of SFT in predicting bioactivity. Moreover, employing molecular dynamics (MD) simulation may help address how released ions can change the surface tension of SBF at the liquid/solid interfaces.

The variations in the pH and ions released from the MBGs were monitored by using color map profiles (Figure 9, Figure 10 and Figure 11). According to the data, a sharp increase in pH and ion release was detectable for up to the first five days, followed by their constant rise for up to 14 days. Then, we observed a decreasing trend for all the groups. The reason behind this observation was attributed to the formation of highly crystalline HAp onto the MBGs (HAp is formed in alkaline pH, and its formation decreases the pH) [39,40]. Furthermore, the re-entering of the doped ions into the structure of newly mineralized HAp could reduce their concentrations in SBF, as also reported previously [16]. As displayed in Figure 9, Figure 10 and Figure 11, the Cu-doped MBGs were more active than their Zn-doped counterparts as regards their behaviors in the biological solutions (faster/higher pH changes and higher ion release). Therefore, it was expected that Cu-doped MBGs were more effective for eliciting biological effects. Concerning the bioactivity results, the incorporation of 7.5 mol% dopants into borate MBGs led to a negligible decrease in the glass bioactivity, as also calculated using the β-based criterion. This might be related to the high bioactivity of mesoporous borate glasses; thereby, mesoporous borate-based MBGs ciykd serve as an excellent platform for the incorporation of higher dosages of therapeutic ions without any significant adverse effect on the bioactivity.

The results of the MTT assay revealed that the synthesized MBGs has no adverse effect on the viability of mouse fibroblasts (L929 cell line) at a concentration of 0.5 mg/mL. However, increasing concentrations of the MBGs (2 and 4 mg/mL) significantly reduced cell viability (Figure 12). It has been reported that borate-based BGs can exert an inhibitory effect on cell survival and proliferation, due to the bursting release of ions from their network to the surrounding environment associated with an increment of pH [16]. It should be noted that these effects could be moderated in in vivo conditions, owing to the large volume of circulating body fluids [41].

Hemolysis is considered to be the disruption of the red blood cell (RBC) membrane that leads to the release of hemoglobin into the surrounding fluids. The hemolysis of RBCs is directly associated with the bio-incompatibility of substances [42]. Figure 13 displays that 2.5% and 7.5 mol% Zn/Cu-doped borate glasses had the lowest hemolysis percentage (5.31 ± 1.63 and 4.30 ± 1.35%, respectively). However, we could not find a reasonable relation between the results obtained from different groups and dopant concentrations.

Since antibacterial activity is one of the most critical features of any implantable material, we investigated the possible effects of the dopant-free and Zn- and Cu-doped MBGs against *P. aeruginosa*. Our results revealed that Zn/Cu-doped borate-based MBGs could significantly reduce bacterial viability in a dose-dependent manner (Figure 14). As previously reported, the antibacterial effects of Zn^2+^ and Cu^2+^ ions were due to the mechanism whereby cell wall/membrane synthesis was inhibited, generating oxidative stress and leading to DNA damage [43]. Compared to Zn-doped MBGs, the incorporation of Cu into the MBGs had higher adverse effects on bacterial viability. One reason for such events was probably the higher activity of Cu-doped MBGs in biological solutions in comparison with Zn-doped counterparts, as discussed above.

## 4. Materials and Methods

### 4.1. Glass Synthesis

The designed nominal compositions of Zn- and Cu-doped 13-93B3 borate glasses are shown in Table 4. The amounts of raw materials, including B(OH)_3_, Ca(NO_3_)_2_·4H_2_O, Zn(NO_3_)_2_·6H_2_O, Cu(NO_3_)·3H_2_O, Mg(NO_3_)_2_·6H_2_O, KNO_3_, NaNO_3_, and (C_2_H_5_)_3_PO_4_ (all from Samchun Chemical Co., Seoul, Republic of Korea) were calculated using HSC chemistry 9.4 (Outotec^®^, ESPOO, Uusimaa, Finland). First, 2 g of P123 (EO20-PO70-EO20, M_W_ = 5800 g/mol) was dissolved in 100 mL of absolute ethanol containing 0.1 mL of hydrochloric acid (HCl, 37% (*w*/*w*)) over 60 min at 70 °C. Then, 100 mL of deionized water was added to the solution. Next, the calculated amounts of the raw materials for synthesizing 10 g borate glasses were separately added to this solution at 60 min intervals. The pH of the final solution was increased to 14 using NH_4_OH (25% (*w*/*w*)), and they were gently shaken for 48 h. The obtained gels were aged for 7 days in sealed bottles. After that, the samples were dried using a two-step process, i.e., 70 °C for 24 h and 140 °C for another 24 h. Finally, the samples were heat-treated at 550 °C for 24 h with a heating rate of 1 °C/min.

### 4.2. Thermodynamic Aspects of BGs

To investigate the thermodynamic aspects of the systems, the data of 23,000 borate glasses, with the maximum molar amount of B_2_O_3_ (out of 423,000 glass compositions), was initially extracted from the SciGlass database version 7.12. Then, the data were classified using SPSS modeler version 18.12 (IBM, Armonk, NY, USA) in accordance with Equation (2):

(2) $$\langle {\mathrm{aB}}_{2}O_{3}-\mathrm{bZnO}-\mathrm{cCuO}-d\;|a=\mathrm{Max},\;b\;\&\;c\;\leq \;10\rangle$$

where a denotes mol% of B_2_O_3_, b and c are mol% of additives/dopants (Zn and Cu, respectively), and d is mol% of other components/additives in the glass structure.

The configurational entropy (S_Conf_) of the classified data was calculated using Equation (3) [28]:

(3) $$S_{\mathrm{Conf}}=-R\sum_{i=1}^{n}X_{i}{\mathrm{lnX}}_{i}$$

where R is the gas constant and X_i_ signifies the molar fraction of elements.

The relation between the calculated S_Conf_ and glass transition temperature (T_g_) was predicted using the SPSS modeler (version 18.22, IBM, Armonk, NY, USA).

### 4.3. Glass Characterizations

The thermal behavior of the glass was studied by differential thermal analysis/thermogravimetric analysis (DTA/TGA) (Bahr STA-503, BAHR, Berlin, Germany). The heating rate of 10 °C/min was applied for all the analyses.

The particle size and surface charge of the MBGs were determined using dynamic light scattering (DLS) analysis (NANO-flex^®^ II, Thermo Fisher Scientific, Waltham, MA, USA). To do this analysis, 0.01 g of MBGs was dispersed in absolute ethanol by applying probe sonication for 10 min.

The mesoporous characteristics of the glass samples were determined using Brunauer–Emmett–Teller (BET) analysis (ASSP 2020, Norcross, GA, USA). In the current analyses, the samples were first degassed at 250 °C for 6 h in a vacuum process. Glass powders also underwent TEM analysis.

X-ray diffraction (XRD) with monochromatic Cu–Kα radiation (D8 Advance, Bruker, Germany), combined with Rietveld analysis, was used for the detection and identification of crystalline phases in the prepared glasses. For this purpose, a step size of 0.02° with a time per step of 1 s was set in the instrument over a 2θ range of 20 to 80°. The Profex package (Graphical Rietveld refinement software, version 5) was used to calculate the lattice constants of the crystallized HAp phase.

The crystallinity degree (Xc) of the mineralized HAp onto the MBGs surface was estimated based on the relation (Equation (4)) reported by Landi et al. [44]:

Xc ≈ 1 − (V_112/300_/I_300_) (4)

where V_112/300_ is the hollow between (112)/(300) reflections, and I_300_ is the intensity of (300) reflection.

The morphology of the BGs before and after soaking in simulated body fluid (SBF) was determined using field-emission scanning electron microscopy (FESEM) (MIRA3, Tescan, Brno, Czech Republic).

### 4.4. Bioactivity Assessment

#### 4.4.1. In Vitro Mineralization

The in vitro mineralization of the synthesized MBGs was evaluated by soaking them in SBF. For this purpose, SBF was first prepared according to the Kokubo’s method [36]. Then, 0.15 g of each glass were immersed in 100 mL of SBF (mass-to-volume ratio 1.5 mg/mL, as suggested by relevant recent studies [45], and shaken for 3 h, 1, 3, 5, 7, 14, and 21 days using an orbital shaker (Stuart Orbital shaker, SI500, London, UK). Changes in the concentration of ions in the solution were recorded via inductively-coupled plasma atomic emission spectroscopy (ICP-AES) analysis.

#### 4.4.2. Tensiometry

A tensiometer (KRUSS-K100, Berlin, Germany) was employed to measure the dynamic surface tension (SFT) of SBF-immersed MBGs at various intervals, including 3 h, 1, 3, 5, 7, 14, 21, and 28 days. In this analysis, the Du Noüy ring approach with a platinum–iridium ring was used. Based on the protocol, 80 mL of SBF (without BG powders) was first added to the instrument’s cell. Then, the ring with a constant speed of 0.1 mm/s was pulled from the medium. The corresponding force (F) to detach the ring from SBF was monitored during the test. The SFT of each medium was calculated as follows (Equation (5)) [46]:

(5) $$\mathrm{SFT}=\frac{F_{\max}}{L*\;\cos ɵ}$$

where F_max_, L, and θ represent the maximum measured force, the ring length, and the contact angle of the medium and ring, respectively.

### 4.5. Biological Assessment

#### 4.5.1. Cell Viability Assay

To study the effects of dopant-free and Zn- and Cu-doped MBGs on cell viability, we performed the MTT assay using mouse fibroblasts (L929 cell line; National Cell Bank, Pasteur Institute of Iran, Tehran, Iran). First, the cells at a concentration of 3 × 10^3^ cells were seeded in 96-well cell culture plates (SPL Lifescience, Pocheon, Republic of Korea) and cultured using Dulbecco’s Modified Eagle Medium (DMEM) enriched with 10% *v*/*v* fetal bovine serum (FBS) and 1% *v*/*v* antibiotic (penicillin/streptomycin) (PAN-Biotech, Minneapolis, MN, USA). Then, the glass samples were added to FBS-free DMEM at concentrations of 0.5, 2, and 4 mg/mL in order to prepare conditioned media. Next, the cells were incubated with the prepared conditioned media for 24, 48, and 72 h. After the incubation times, the MTT ([3-(4,5-dimethyl-2-thiazolyl)-2,5-diphenyl tetrazolium bromide]) solution with the final concentration of 0.5 mg/mL was added to each well and incubated for another 4 h. After that, all culture media were gently removed and replaced with 100 mL of dimethyl sulfoxide (DMSO, Sigma-Aldrich, St. Louis, MO, USA) and incubated for 20 min. Finally, cell viability was determined by measuring the absorbance of each well at 570 nm and 690 nm by using a microplate reader (Epoch, Bio Tek, Westmont, IL, USA).

#### 4.5.2. Blood Compatibility Test

To evaluate the blood compatibility of the synthesized MBGs, whole blood was initially collected from a consenting adult woman and treated with sodium citrate as an anticoagulant agent. Then, the glass samples (4 mg/mL) were added to the whole blood and incubated at 37 °C for 60 min. Blood diluted in deionized water and normal saline were considered positive and negative controls, respectively. Afterward, the samples were centrifuged at 1500 rpm for 10 min, and the absorbance of the supernatant was read at 545 nm by using a microplate reader (Epoch, BioTek, Shoreline, WA, US). The amount of hemolysis was calculated via the following formula (Equation (6)) [47]:

(6) $$\mathrm{Hemolysis}\;(\%)=[\frac{D_{s}-{\;D}_{n}}{D_{p}-{\;D}_{n}}]\times 100$$

where D_s_, D_p_, and D_n_ represent the absorption of samples, positive control, and negative control, respectively.

#### 4.5.3. Antibacterial Activities

Antibacterial activity of the dopant-free and Zn- and Cu-doped MBGs were studied against *Pseudomonas aeruginosa* (*P. aeruginosa*) according to the literature [48]. For this purpose, the bacteria were cultured in a lysogeny broth (LB) medium at 37 °C. After the sterilization, the MBGs (2 and 4 mg/mL) were added to LB and incubated at 37 °C for 24 h. Then 1–5 × 10^5^ CFU/mL of the bacterial suspension was added to the glass-containing media and incubated at 37 °C for 24 h. The samples without the glasses were considered the positive control. The antibacterial properties of the MBGs were calculated based on the absorption of a bacterial suspension at 600 nm with a microplate reader (Epoch, Bio Tek, US). The viability of the bacteria was calculated using Equation (7) [49]:

(7) $${\mathrm{Antibacterial}\;\mathrm{activity}\;(\%)=(D}_{s}{/D}_{p})\;\times \;100$$

where D_s_ denotes the absorption of the glass-treated bacteria, and D_p_ represents the adsorption of the positive control.

### 4.6. Statistical Analysis

The characterization data, including thermal, DLS, and BET analyses, were repeated three times, and the obtained results were expressed as mean ± SD; the data was classified using K-means algorithms (SPSS modular, version 18.22, IBM, Armonk, NY, USA). The SFT test was repeated five times, and the results were reported as mean ± SD. The SFT data were classified using SPSS modular by the K-means clustering algorithm and chi-square automatic interaction detection (CHAID) tree. The biological experiments, including the cell viability assay, the blood compatibility assay, and the antibacterial activity test, were performed three times, and the results were reported as mean ± SD. The results were then analyzed using GraphPad Prism (version 9.4.1(681), San Diego, CA, USA) with a one-way ANOVA variance model followed by post-hoc analysis. The significant data were marked based on their *p*-values, including * *p* ≤ 0.05, ** *p* ≤ 0.01, *** *p* ≤ 0.001, and **** *p* ≤ 0.0001.

## 5. Conclusions

In the current study, we successfully synthesized a series of Zn- and Cu-doped 13-93B3 borate glasses by a modified sol–gel method (using nitrate precursors) in the presence of Pluronic P123. The characterization results were systematically classified using data science algorithms, which can be useful for drawing a reasonable strategy for the future of the field. A negative linear relation of T_g_ and S_Conf_ (a variable for measuring the disorder of a thermodynamic system) was observed in the doped MBGs; the increase in the number and concentration of dopants enhanced the S_Conf_ and reduced the T_g_. Using the data science algorithms, our data (13 samples) were divided into five similar clusters, including a cluster for one un-doped sample and four clusters for the doped ones, indicating the significant role of the dopants. According to the K-means clustering algorithm, the most important independent groups were the doped MBGs with 1 and 5 mol% of dopants. In addition, we introduced a new possible reason for the mineralization process of HAp on the glass surface related to changes in the SFT of SBF. According to the K-means algorithm and CHAID tree, we found that SFT mainly changed in the period of 3 h, 1 day, 3 days, 5 days, and 7 days. Further, based on the variation of SFT, we introduced a quantitative number (β) for evaluating bioactivity. The in vitro mineralization studies indicated the negligible effect of dopants on the bioactivity of borate glasses. The synthesized doped glasses at a concentration of 0.5 mg/mL had no adverse effects on the viability of fibroblasts and showed suitable antibacterial activity. In conclusion, this type of glass can be considered a useful substance for skin wound healing applications; however, in vivo animal studies should be conducted in the future to obtain more data close to human body conditions.

## Figures and Tables

**Table 1:** The results of thermal, DLS, and BET analyses.

Sample	T_g_ (°C)	Particle Size (nm)	Zeta Potential (mV)	S_BET_ (m^2^/g)	Cluster
B0	773 ± 6	378 ± 27	−(12 ± 4)	54 ± 2	1
Zn1	664 ± 9	209 ± 13	−(18 ± 4)	68 ± 7	3
Zn2.5	630 ± 7	166 ± 14	−(18 ± 3)	79 ± 6	3
Zn5	590 ± 13	114 ± 9	−(19 ± 4)	98 ± 4	4
Zn7.5	566 ± 6	92 ± 6	−(19 ± 3)	123 ± 8	4
Cu1	695 ± 9	217 ± 14	−(21 ± 4)	66 ± 9	3
Cu2.5	684 ± 4	145 ± 13	−(20 ± 4)	76 ± 8	3
Cu5	680 ± 6	112 ± 6	−(19 ± 4)	93 ± 6	2
Cu7.5	676 ± 9	96 ± 7	−(20 ± 4)	122 ± 9	2
Zn/Cu1	660 ± 11	193 ± 6	−(22 ± 4)	69 ± 5	3
Zn/Cu2.5	620 ± 10	151 ± 7	−(24 ± 4)	95 ± 4	5
Zn/Cu5	560 ± 7	104 ± 7	−(26 ± 5)	101 ± 3	2
Zn/Cu7.5	556 ± 9	64 ± 6	−(23 ±5)	131 ± 4	2

**Table 2:** The calculated crystallinity degrees and lattice constants of the MBGs after incubation in SBF for three weeks.

	Zn			Cu			Zn/Cu		
Dopant (mol%)	Crystallinity Degree (%)	a (Å)	c (Å)	Crystallinity Degree (%)	a (Å)	c (Å)	Crystallinity Degree (%)	a (Å)	c (Å)
0	80	9.424	6.879	80	9.242	6.879	80	9.242	6.879
1	76	9.435	6.882	74	9.434	6.881	71	9.436	6.882
2.5	70	9.44	6.884	69	9.439	6.884	67	9.441	6.883
5	66	9.44	6.887	64	9.439	6.886	62	9.441	6.884
7.5	65	9.441	6.890	63	9.441	6.888	60	9.446	6.891

**Table 3:** The analysis of the bioactivity number (β) and its normalized values in different groups.

Sample	Bioactivity Number (β)	Normalized β	Normalized β Compared to B0 (%)
B0	0.774074	1.000000	0.0
Zn1	0.812963	1.050239	5.0
Zn2.5	0.811111	1.047847	4.8
Zn5	0.716059	0.925053	−7.5
Zn7.5	0.713571	0.921838	−7.8
Cu1	0.809925	1.046315	4.6
Cu2.5	0.795630	1.027847	2.8
Cu5	0.705556	0.911484	−8.8
Cu7.5	0.700000	0.904306	−9.6
Zn/Cu1	0.911777	1.177894	17.8
Zn/Cu2.5	0.891925	1.152248	15.2
Zn/Cu5	0.798148	1.031100	3.1
Zn/Cu7.5	0.792593	1.023924	2.4

**Table 4:** The nominal compositions of the dopant-free (B0) and Zn- and Cu-doped 13-93B3 MBGs (mol%).

Sample	B_2_O_3_	CaO	ZnO	CuO	MgO	K_2_O	Na_2_O	P_2_O_5_
B0	54.6	22.1	0	0	7.6	7.9	6.1	1.7
Zn1	54.6	21.1	1	0	7.6	7.9	6.1	1.7
Zn2.5	54.6	19.6	2.5	0	7.6	7.9	6.1	1.7
Zn5	54.6	17.1	5	0	7.6	7.9	6.1	1.7
Zn7.5	54.6	14.6	7.5	0	7.6	7.9	6.1	1.7
Cu1	54.6	21.1	0	1	7.6	7.9	6.1	1.7
Cu2.5	54.6	19.6	0	2.5	7.6	7.9	6.1	1.7
Cu5	54.6	17.1	0	5	7.6	7.9	6.1	1.7
Cu7.5	54.6	14.6	0	7.5	7.6	7.9	6.1	1.7
Zn/Cu1	54.6	21.1	0.5	0.5	7.6	7.9	6.1	1.7
Zn/Cu2.5	54.6	19.6	1.25	1.25	7.6	7.9	6.1	1.7
Zn/Cu5	54.6	17.1	2.5	2.5	7.6	7.9	6.1	1.7
Zn/Cu7.5	54.6	14.6	3.75	3.75	7.6	7.9	6.1	1.7

## Data Availability

Data available in the study.
